# A real-world disproportionality analysis of mesalazine data mining of the public version of FDA adverse event reporting system

**DOI:** 10.3389/fphar.2024.1290975

**Published:** 2024-01-31

**Authors:** Mingdi Liu, Liting Gu, Yuning Zhang, Honglan Zhou, Yishu Wang, Zhi-Xiang Xu

**Affiliations:** ^1^ Key Laboratory of Pathobiology, Ministry of Education, Jilin University, Changchun, Jilin, China; ^2^ Department of Urology, The First Hospital of Jilin University, Changchun, Jilin, China

**Keywords:** mesalazine, adverse event, FAERS, pharmacovigilance, data mining

## Abstract

**Background:** Mesalazine, a preparation of 5-aminosalicylic acid, is a medication widely used in clinical practice as a first-line therapy in the treatment of mild and moderate inflammatory bowel disease. However, the long-term safety of mesalazine in large sample population was unknown. The current study was to assess mesalazine -related adverse events of real-world through data mining of the US Food and Drug Administration Adverse Event Reporting System (FAERS).

**Methods:** Disproportionality analyses, including the reporting odds ratio (ROR), the proportional reporting ratio the Bayesian confidence propagation neural network and the multi-item gamma Poisson shrinker (MGPS) algorithms were employed to quantify the signals of mesalazine -associated AEs.

**Results:** Out of 14,149,980 reports collected from the FDA Adverse Event Reporting System database, 24,284 reports of mesalazine -associated AEs were identified. A total of 170 significant disproportionality preferred terms conforming to the four algorithms simultaneously were retained. The most common AEs included colitis ulcerative, diarrhoea, condition aggravated, crohn’s disease, fatigue, abdominal pain, nausea, haematochezia, which were corresponding to those reported in the specification and clinical trials. Unexpected significant AEs as dizziness, drug ineffective, drug hypersensitivity, infection, off label use, weight decreased, decreased appetite, arthralgia, rash might also occur. The median onset time of mesalazine -related AEs was 1,127 days (interquartile range [IQR] 1,127–1,674 days), and most of the cases occurred 2 years later (n = 610, 70.93%) and within the first 1 month (n = 89, 10.35%) after mesalazine initiation.

**Conclusion:** Results of our study were consistent with clinical observations. We also found potential new and unexpected AEs signals for mesalazine, suggesting prospective clinical studies were needed to confirm these results and illustrate their relationship. Our results could provide valuable evidence for further safety studies of mesalazine.

## 1 Introduction

The prevalence of inflammatory bowel disease (IBD), encompassing ulcerative colitis (UC) and Crohn’s disease (CD), is increasing worldwide, and new treatments for IBD (including biologics) have emerged in recent years (Ford et al., 2011a). Among the first-line treatments for IBD are 5-aminosalicylic acid derivatives (5-ASAs) especially mesalazine, which is effective in inducing and maintaining remission in most cases of UC with mild or moderate activity (Veloso et al., 2021; Suzuki et al., 2022).

Mesalazine (mesalamine), also known chemically as 5-aminosalicylic acid (5-ASA), is commonly used in the treatment of UC (Ford et al., 2012). Mesalazine exerts an anti-inflammatory effect on the intestinal wall after taken orally (Ford et al., 2011b). Because of the anti-ulcer and antioxidant efficacy, mesalazine is not only used in the treatment of UC, but also in other diseases (Beiranvand and Bahramikia, 2020; Li et al., 2022). Some studies have shown that mesalazine also improve mucosal healing and reduced risk of colorectal cancer, and that it halves the risk of colitis-associated cancer or dysplasia in patients with UC (Bonovas et al., 2017). Mesalazine, a non-steroidal anti-inflammatory drug (NSAIDs), has an anti-inflammatory effect that has not been fully explained; however, the available data suggest that it antagonises pro-inflammatory mediators such as NF-κB, γ-IFN, IL-8 and TNF-α. It also inhibits the cyclo-oxygenase (COX) and lipoxygenase (LOX) pathways, which inhibit the release of the inflammation-associated prostaglandins E2 and leukotriene. There is also an anti-inflammatory mechanism of action suggesting that mesalazine increases PPARγ expression in gastrointestinal epithelial cells. The available data suggest that mesalazine may also inhibit the proliferation of tumor cells through a number of pathways (Słoka et al., 2023). Mesalazine is the pharmacologically active ingredient in sulfasalazine, which was the first compound used in the treatment of ulcerative colitis. Sulfasalazine, on the other hand, is the inactive ingredient in sulfasalazine and can cause serious side effects. For example, it sometimes produces symptoms such as fever, diarrhoea and bloody stools (Matsumoto and Mashima, 2020).

The FDA Adverse Event Reporting System (FAERS) database is a publicly accessible spontaneous reporting system, which covers tens of millions of case reports of adverse drug events (ADEs) submitted by physicians, pharmacists, manufacturers, and others (Cohen et al., 2000; Rahimi et al., 2009). The FAERS is currently the world’s largest pharmacovigilance database and is an effective tool for detecting ADRs associated with drug exposure (Shu et al., 2022a; Shu et al., 2022b). In the present study, we retrospectively analyzed the AEs reported from January 2018 to December 2022 with mesalazine through data mining of FAERS.

## 2 Materials and methods

### 2.1 Study design and data sources

FAERS, as a well-known publicly available post-marketing safety surveillance database, which researchers collect AEs reports by health professionals, pharmaceutical manufacturers, individual patients and others. The FAERS data files contained seven types of datasets: patient demographic and administrative information (DEMO), drug information (DRUG), coded for the adverse events (REAC), patient outcomes (OUTC), report sources (RPSR), therapy start dates and end dates for reported drugs (THER), and indications for drug administration (INDI), and deleted cases. All the data downloaded from the U.S. FDA website were imported into MySQL 8.0 for further analysis. This research based on FAERS database, extracting data from January 2018 to December 2022.

**FIGURE 1:**
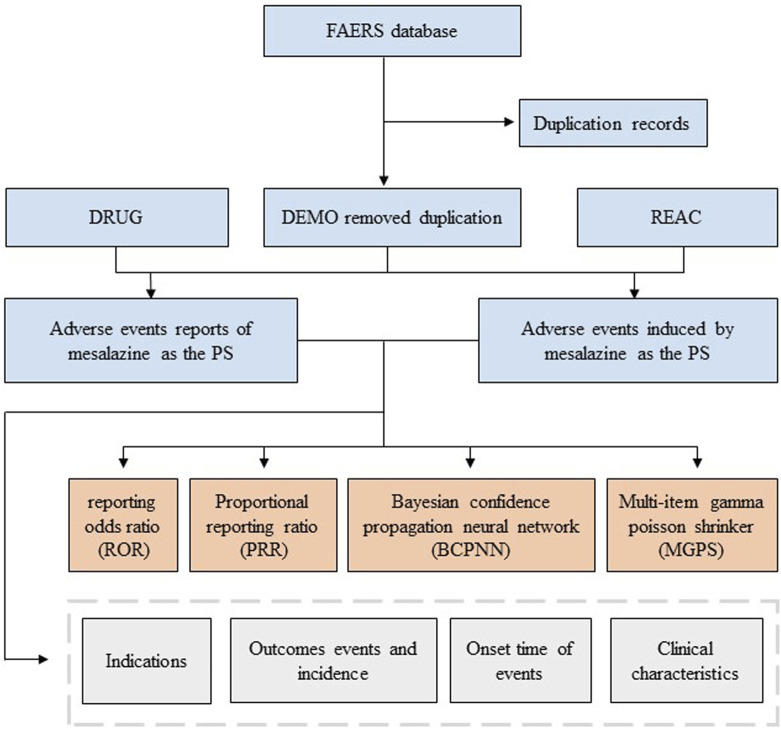
Flowchart of identifying adverse event cases of mesalazine and statins from the FAERS database.

AEs in the FAERS database were coded by Medical Dictionary for Regulatory Activities 24.0 (MedDRA). The structural hierarchy of the MedDRA terminology was divided into five levels: system organ class (SOC), high-level group term (HLGT), high-level term (HLT), preferred term (PT), and lowest-level term (LLT) (Burr et al., 2019). All AEs of mesalazine reports taken from the REAC files in the FAERS database were identified to describe the frequency and intensity based on MedDRA at SOC and PT levels in our study (Cammà et al., 1997). The reported drugs in FAERS were categorized into four patterns: PS (primary suspect), SS (secondary suspect), C (concomitant), and I (interacting). Serious patient outcomes were defined as death (DE), life-threatening (LT), hospitalization-initial or prolonged (HO), disability (DS), congenital anomaly (CA) or other important medical event (OT) (Steinhart et al., 1994). Clinical characteristics including gender, age, reporting area, reporter, reporting time and outcomes of patients with mesalazine -related AEs were collected (Louis et al., 2022) (Figure 1).

### 2.2 Statistical analysis

Descriptive analysis was used to show the characteristics of all AE reports regarding to mesalazine. Disproportionality analysis, which is widely used in pharmacovigilance study, was performed to identify potential signals between mesalazine and all AEs in our investigation. Reporting odds ratio (ROR), the proportional reporting ratio (PRR), the Bayesian confidence propagation neural network (BCPNN), and the multi-item gamma Poisson shrinker (MGPS) are four major specific indices that were calculated using standard formulas to assess potential associations between mesalazine and AEs as presented in Table 1. Only those signals with at least three target AE records to target drugs were calculated in our study. At least one of the four algorithms meets the criteria should be considered as a positive signal of drug-associated AEs (lower limit of 95% CI > 1, N ≥ 3; PRR≥2, χ^2^ ≥ 4, N ≥ 3; IC025 > 0 or EBGM05 > 2). In this study, we selected AE signals that simultaneously met all of the above four algorithm standards for research (Shu et al., 2022a; Shu et al., 2022b; Hou et al., 2022).

**TABLE 1:** Four major algorithms used for signal detection.

Algorithms	Equation	Criteria
ROR	ROR = ad/b/c	lower limit of 95% CI > 1, N ≥ 3
	95%CI = e^ln(ROR)±1.96(1/a+1/b+1/c+1/d)^0.5^	
PRR	PRR = a(c + d)/c/(a+b)	PRR≥2, χ^2^ ≥ 4, N ≥ 3
	χ^2^ = [(ad-bc)^2](a+b + c + d)/[(a+b)(c + d)(a+c)(b + d)]	
BCPNN	IC = log_2_a(a+b + c + d)(a+c)(a+b)	IC025 > 0
	95%CI = E(IC) ± 2V(IC)^0.5	
MGPS	EBGM = a(a+b + c + d)/(a+c)/(a+b)	EBGM05 > 2
	95%CI = e^ln(EBGM)±1.96(1/a+1/b+1/c+1/d)^0.5^	

**Notes:** Equation: a, number of reports containing both the target drug and target adverse drug reaction; b, number of reports containing other adverse drug reaction of the target drug; c, number of reports containing the target adverse drug reaction of other drugs; d, number of reports containing other drugs and other adverse drug reactions.

**Abbreviations**: 95% CI, 95% confidence interval; N, the number ofreports; χ2, chi-squared; IC, information component; IC025, the lower limit of 95% CI, of the IC; E(IC), the IC, expectations; V(IC), the variance of IC; EBGM, empirical Bayesian geometric mean; EBGM05, the lower limit of 95% CI, of EBGM. ROR, reporting odds ratio; PRR, proportional reporting ratio; BCPNN, bayesian confidence propagation neural network; MGPS, multi-item gamma Poisson shrinker.

The time to onset and serious outcome probability of AEs were calculated. The onset time is defined as the interval between EVENT_DT (date of AE occurrence) and START_DT (start date for mesalazine use). In addition, reports with input errors (EVENT_DT earlier than START_DT), inaccurate date entries and missing specific data were excluded. Severe outcomes mainly included life threatening events or those causing hospitalization, disability, or death. Moreover, reports with serious outcome events attributed to drug toxicity were counted, and the proportion was calculated as dividing the number of serious outcomes by the total number of reported events. All data processing and statistical analyses were performed using MYSQL 8.0, Navicat Premium 15, Microsoft EXCEL 2019 and the GraphPad Prism 8 (GraphPad Software, CA, United States) (Li et al., 2021; Shu et al., 2022a; Shu et al., 2022b).

## 3 Results

### 3.1 General characteristics

From January 2018 to December 2022, a total of 14,149,980 AE reports submitted to FAERS database, among which 24,284 reports on mesalazine were reported. The characteristics of AE reports submitted for mesalazine are described in Table 2. The number of reported AEs was variable from 2018 to 2022, the most reported year was 2021 (14.46%), followed by 2018 (12.44%). Among all reports, females (50.34%) accounted for a larger proportion than males (39.32%). The largest percentages of reports (2.24%) were in elderly individuals (aged >68 years) and patients aged 18–28 years also accounted for a high proportion with 2.19% (n = 531), followed by aged 28–38 years (2.10%). Colitis ulcerative was the most reported indication (17.20%), followed by crohn’s disease (6.54%), colitis (1.89%), inflammatory bowel disease (0.85%). Most of reports were came from United States (29.90%), followed by Canada (18.99%), Japan (6.38%), United Kindom (4.43%), and Italy (2.60%), mainly submitted by health professionals (39.60%) and Consumers (33.81%). Mesalazine was the primary suspect (30.97%) in most reports. Hospitalization-initial or prolonged (HO) (7.95%) was the most frequently reported serious outcome, and death or life-threatening events were reported in 223 (0.92%) and 164 (0.68%) cases respectively.

**TABLE 2:** Clinical characteristics of reports with mesalazine from the FAERS database.

Characteristics		Case Number, n	Case proportion, %
	Number of events	24,284	
**Gender**	female	12,224	50.34
	male	9,549	39.32
	unknown	2,511	10.34
**Age(years)**	<18	400	1.65
	18–28	531	2.19
	28–38	511	2.10
	38–48	400	1.65
	48–58	440	1.81
	58–68	433	1.78
	>68	545	2.24
	unknown	21,024	86.58
**Indications (top five)**	product used for unknown indication	6,465	26.62
	colitis ulcerative	4,178	17.20
	crohn’s disease	1,589	6.54
	colitis	459	1.89
	inflammatory bowel disease	206	0.85
**Serious outcome**	Death (DE)	223	0.92
	life-threatening (LT)	164	0.68
	hospitalization-initial or prolonged (HO)	1930	7.95
	Disability (DS)	124	0.51
	congenital anomaly (CA)	41	0.17
**Reported countries (top five)**	Japan	1,550	6.38
	United States	7,262	29.90
	Canada	4,611	18.99
	United Kindom	1,075	4.43
	Italy	632	2.60
**Reporting year**	2022	2,561	10.55
	2021	3,512	14.46
	2020	1,637	6.74
	2019	2,168	8.93
	2018	3,021	12.44
**Reported Person**	Health profession	9,616	39.60
	Consumer	8,211	33.81
	Unknown	6,457	26.59
**Role code**	primary suspect (PS)	7,521	30.97
	secondary suspect (SS)	6,029	24.83
	concomitant (C)	5,421	22.32
	Interacting (I)	81	0.33

### 3.2 Signal detection

A total of 170 significant PTs of interest conforming to all of the four algorithms simultaneously are described in Table 3. In this study, anaemia (PT: 10002034), colitis ulcerative, diarrhoea, crohn’s disease, abdominal pain, nausea (PT: 10028813), infection, maternal exposure during pregnancy, foetal exposure during pregnancy were present, which consistent with the instructions and medication warnings. Of note, unexpected significant AEs, including Dizziness, Drug hypersensitivity, Infection, Weight decreased, Decreased appetite, Arthralgia, Headache, Dyspnoea, Rash and so on, were uncovered in the label.

**TABLE 3:** Signal Strength of AEs of mesalazine at the System Organ Class (SOC) Level in FAERS Database.

System organ class (SOC)	Preferred terms	PT/N	ROR (95% two-sided CI)	PRR (95% two-sided CI)	χ^2^	IC (IC025)	EBGM (EBGM05)
Blood and lymphatic system disorders	Anaemia	378	2.16 (1.95–2.4)	2.14 (1.94–2.37)	230.57	1.1 (0.76)	2.14 (1.93)
	Pancytopenia	157	3.11 (2.66–3.64)	3.1 (2.65–3.62)	219.72	1.62 (1.1)	3.08 (2.63)
	Thrombocytopenia	141	1.35 (1.15–1.6)	1.35 (1.14–1.59)	12.47	0.43 (−0.12)	1.35 (1.14)
	Leukopenia	126	2.72 (2.28–3.24)	2.71 (2.28–3.23)	133.99	1.43 (0.85)	2.7 (2.27)
Cardiac disorders	Dizziness	555	1.05 (0.96–1.14)	1.05 (0.97–1.14)	1.19	0.07 (−0.22)	1.05 (0.96)
	Chest pain	310	1.57 (1.4–1.75)	1.56 (1.4–1.74)	61.93	0.64 (0.26)	1.56 (1.39)
	Oedema peripheral	218	1.6 (1.4–1.83)	1.6 (1.4–1.82)	48.3	0.67 (0.23)	1.6 (1.4)
	Myocarditis	191	21.13 (18.26–24.45)	20.97 (18.15–24.24)	3,446.53	4.33 (3.84)	20.05 (17.33)
	Pericarditis	162	16.51 (14.11–19.33)	16.41 (14.03–19.19)	2,244.33	3.99 (3.46)	15.84 (13.54)
	Tachycardia	147	1.63 (1.39–1.92)	1.63 (1.39–1.92)	35.17	0.7 (0.16)	1.63 (1.38)
	Chest discomfort	143	1.49 (1.27–1.76)	1.49 (1.27–1.76)	22.65	0.57 (0.03)	1.49 (1.26)
	Palpitations	139	1.1 (0.93–1.3)	1.1 (0.93–1.3)	1.24	0.14 (−0.41)	1.1 (0.93)
	Syncope	132	1.24 (1.04–1.47)	1.24 (1.04–1.47)	5.74	0.31 (−0.26)	1.24 (1.04)
	Pericardial effusion	100	4.51 (3.7–5.49)	4.49 (3.69–5.47)	265.62	2.16 (1.5)	4.46 (3.66)
Eye disorders	Macular degeneration	247	22.25 (19.57–25.31)	22.04 (19.41–25.03)	4,701.78	4.39 (3.96)	21.02 (18.48)
Gastrointestinal disorders	Colitis ulcerative	1877	80.32 (76.34–84.49)	74.18 (70.76–77.78)	115,710.47	5.99 (5.82)	63.45 (60.31)
	Diarrhoea	1,537	2.64 (2.51–2.78)	2.54 (2.42–2.67)	1,461.13	1.34 (1.17)	2.53 (2.4)
	Crohn’s disease	1,307	33.18 (31.32–35.15)	31.45 (29.78–33.22)	35,951.81	4.88 (4.68)	29.38 (27.74)
	Abdominal pain	1,167	5.58 (5.26–5.92)	5.36 (5.07–5.67)	4,121.64	2.41 (2.21)	5.31 (5)
	Nausea	1,046	1.29 (1.21–1.37)	1.28 (1.2–1.36)	64.98	0.35 (0.14)	1.28 (1.2)
	Haematochezia	975	23.15 (21.68–24.72)	22.26 (20.9–23.71)	18,839.6	4.41 (4.19)	21.22 (19.87)
	Vomiting	691	1.47 (1.36–1.58)	1.46 (1.35–1.57)	99.56	0.54 (0.29)	1.45 (1.35)
	Frequent bowel movements	498	28.9 (26.38–31.68)	28.33 (25.9–30.99)	12,305.1	4.74 (4.43)	26.65 (24.32)
	Colitis	477	16.07 (14.65–17.62)	15.77 (14.41–17.27)	6,361.13	3.93 (3.62)	15.25 (13.91)
	Rectal haemorrhage	448	10.95 (9.96–12.03)	10.76 (9.81–11.81)	3,867.47	3.4 (3.08)	10.52 (9.57)
	Abdominal pain upper	402	1.97 (1.79–2.18)	1.96 (1.78–2.16)	188.29	0.97 (0.64)	1.95 (1.77)
	Abdominal distension	334	3.45 (3.1–3.85)	3.42 (3.07–3.81)	567.25	1.77 (1.4)	3.4 (3.05)
	Pancreatitis	311	4.84 (4.33–5.42)	4.79 (4.29–5.35)	921.57	2.25 (1.87)	4.75 (4.24)
	Abdominal discomfort	245	1.47 (1.3–1.67)	1.47 (1.3–1.66)	36.16	0.55 (0.13)	1.47 (1.29)
	Constipation	233	1.2 (1.05–1.36)	1.19 (1.05–1.36)	7.17	0.25 (−0.18)	1.19 (1.05)
	Oropharyngeal pain	226	2.74 (2.4–3.13)	2.72 (2.39–3.1)	244.29	1.44 (1)	2.71 (2.38)
	Diarrhoea haemorrhagic	222	28.12 (24.54–32.23)	27.87 (24.35–31.91)	5,379.2	4.71 (4.26)	26.24 (22.9)
	Flatulence	202	3.66 (3.19–4.21)	3.64 (3.17–4.18)	381.83	1.86 (1.39)	3.62 (3.15)
	Gastrointestinal disorder	161	1.99 (1.71–2.33)	1.99 (1.7–2.32)	77.91	0.99 (0.47)	1.98 (1.7)
	Pancreatitis acute	155	6.8 (5.8–7.97)	6.76 (5.77–7.92)	744.28	2.74 (2.21)	6.67 (5.69)
	Mucous stools	145	55.93 (47.03–66.52)	55.6 (46.79–66.07)	6,839.67	5.63 (5.05)	49.37 (41.51)
	Intestinal obstruction	136	4.31 (3.64–5.1)	4.29 (3.63–5.08)	336.99	2.09 (1.53)	4.26 (3.59)
	Dysphagia	133	1.36 (1.14–1.61)	1.35 (1.14–1.6)	11.98	0.44 (−0.13)	1.35 (1.14)
	Defaecation urgency	110	25.58 (21.1–31.02)	25.47 (21.02–30.86)	2,419.36	4.59 (3.95)	24.11 (19.88)
	Gastrooesophageal reflux disease	110	1.37 (1.14–1.66)	1.37 (1.14–1.65)	10.73	0.46 (−0.16)	1.37 (1.14)
	Dyspepsia	109	1.09 (0.9–1.31)	1.09 (0.9–1.31)	0.7	0.12 (−0.5)	1.09 (0.9)
	Inflammatory bowel disease	107	8.23 (6.8–9.97)	8.2 (6.77–9.92)	657.01	3.01 (2.38)	8.06 (6.66)
	Gastrointestinal haemorrhage	101	0.94 (0.78–1.15)	0.94 (0.78–1.15)	0.27	−0.08 (−0.73)	0.95 (0.78)
General disorders and administration site conditions	Drug ineffective	2,761	1.92 (1.84–1.99)	1.81 (1.75–1.88)	1,064.34	0.85 (0.72)	1.81 (1.74)
	Condition aggravated	1,379	5.37 (5.09–5.68)	5.13 (4.87–5.4)	4,572.91	2.34 (2.16)	5.08 (4.81)
	Fatigue	1,205	1.62 (1.53–1.72)	1.59 (1.5–1.68)	268.71	0.66 (0.47)	1.59 (1.5)
	Pyrexia	965	3.11 (2.91–3.32)	3.02 (2.84–3.22)	1,314.17	1.59 (1.37)	3.01 (2.82)
	Malaise	709	1.47 (1.36–1.58)	1.45 (1.35–1.56)	101.86	0.54 (0.29)	1.45 (1.35)
	Pain	691	0.96 (0.89–1.03)	0.96 (0.89–1.03)	1.12	−0.06 (−0.31)	0.96 (0.89)
	Injection site pain	568	1.85 (1.7–2.01)	1.83 (1.68–1.98)	213.35	0.87 (0.59)	1.82 (1.68)
	Asthenia	499	1.39 (1.27–1.52)	1.38 (1.27–1.51)	53.49	0.47 (0.17)	1.38 (1.27)
	Drug intolerance	291	3 (2.68–3.37)	2.98 (2.66–3.34)	379.99	1.57 (1.18)	2.97 (2.64)
	No adverse event	267	1.44 (1.27–1.62)	1.43 (1.27–1.61)	34.42	0.52 (0.11)	1.43 (1.27)
	Feeling abnormal	250	0.89 (0.78–1.01)	0.89 (0.79–1.01)	3.38	−0.17 (−0.59)	0.89 (0.79)
	Death	248	0.21 (0.18–0.24)	0.22 (0.19–0.25)	727.47	−2.19 (−2.61)	0.22 (0.19)
	Drug interaction	233	1.38 (1.21–1.57)	1.37 (1.21–1.56)	23.39	0.46 (0.03)	1.37 (1.21)
	Treatment failure	203	2.32 (2.02–2.66)	2.31 (2.01–2.65)	148.62	1.2 (0.74)	2.3 (2)
	Therapeutic product effect decreased	176	5.67 (4.88–6.58)	5.64 (4.86–6.53)	658.55	2.48 (1.98)	5.57 (4.8)
	Therapeutic product effect incomplete	166	2.79 (2.39–3.25)	2.78 (2.38–3.23)	186.15	1.47 (0.96)	2.76 (2.37)
	Gait disturbance	144	0.74 (0.63–0.87)	0.74 (0.63–0.87)	12.63	−0.43 (−0.97)	0.74 (0.63)
	Influenza like illness	142	1.51 (1.28–1.78)	1.51 (1.28–1.78)	23.9	0.59 (0.04)	1.51 (1.28)
	General physical health deterioration	137	1.41 (1.19–1.66)	1.4 (1.19–1.66)	15.51	0.49 (−0.07)	1.4 (1.19)
	Inflammation	134	3.3 (2.78–3.91)	3.29 (2.77–3.89)	209.76	1.71 (1.14)	3.27 (2.76)
	Adverse drug reaction	120	1.26 (1.06–1.51)	1.26 (1.06–1.51)	6.27	0.33 (−0.26)	1.26 (1.05)
	Peripheral swelling	118	0.93 (0.78–1.12)	0.93 (0.78–1.12)	0.52	−0.1 (−0.7)	0.93 (0.78)
	Flushing	113	1.02 (0.85–1.23)	1.02 (0.85–1.23)	0.03	0.03 (−0.58)	1.02 (0.85)
	Injection site pruritus	113	1.59 (1.32–1.91)	1.58 (1.32–1.9)	23.73	0.66 (0.05)	1.58 (1.31)
	Injection site swelling	103	1.26 (1.04–1.53)	1.26 (1.04–1.53)	5.28	0.33 (−0.31)	1.26 (1.04)
	Therapeutic response shortened	102	6.47 (5.32–7.87)	6.45 (5.3–7.83)	457.37	2.67 (2.02)	6.37 (5.23)
Hepatobiliary disorders	Drug-induced liver injury	102	3.71 (3.06–4.52)	3.7 (3.05–4.5)	197.14	1.88 (1.24)	3.68 (3.03)
Immune system disorders	Drug hypersensitivity	370	1.35 (1.22–1.49)	1.34 (1.21–1.49)	32.25	0.42 (0.08)	1.34 (1.21)
	Hypersensitivity	292	1.53 (1.36–1.72)	1.52 (1.36–1.71)	52.54	0.61 (0.22)	1.52 (1.36)
	Infusion related reaction	279	7.68 (6.82–8.65)	7.6 (6.76–8.55)	1,567.18	2.9 (2.51)	7.49 (6.65)
	Urticaria	205	1.23 (1.07–1.41)	1.23 (1.07–1.41)	8.65	0.3 (−0.16)	1.23 (1.07)
	Psoriasis	167	1.3 (1.12–1.52)	1.3 (1.12–1.52)	11.5	0.38 (−0.13)	1.3 (1.12)
	Interstitial lung disease	135	3.08 (2.6–3.65)	3.07 (2.59–3.63)	185.38	1.61 (1.05)	3.06 (2.58)
Infections and infestations	Infection	195	1.47 (1.27–1.69)	1.46 (1.27–1.68)	27.96	0.55 (0.08)	1.46 (1.27)
	Sepsis	178	1.71 (1.48–1.98)	1.71 (1.47–1.97)	51.18	0.77 (0.28)	1.7 (1.47)
	*Clostridium difficile* infection	161	11.61 (9.92–13.58)	11.54 (9.87–13.48)	1,499.43	3.49 (2.97)	11.26 (9.62)
	Influenza	126	1.38 (1.16–1.65)	1.38 (1.16–1.64)	12.84	0.46 (−0.12)	1.38 (1.16)
	Herpes zoster	106	1.99 (1.64–2.41)	1.98 (1.64–2.4)	50.66	0.99 (0.35)	1.98 (1.64)
Injury, poisoning and procedural complications	Off label use	2,253	3.3 (3.15–3.44)	3.08 (2.96–3.21)	3,241.85	1.62 (1.47)	3.07 (2.94)
	Inappropriate schedule of product administration	473	4.17 (3.8–4.57)	4.11 (3.75–4.49)	1,103.5	2.03 (1.72)	4.08 (3.72)
	Incorrect dose administered	424	2 (1.82–2.21)	1.99 (1.81–2.18)	207.72	0.99 (0.66)	1.98 (1.8)
	Product use issue	404	2.19 (1.99–2.42)	2.17 (1.97–2.4)	255.52	1.12 (0.79)	2.17 (1.96)
	Intentional product use issue	393	5.73 (5.18–6.34)	5.66 (5.12–6.24)	1,486.29	2.48 (2.15)	5.6 (5.06)
	Product use in unapproved indication	264	1.2 (1.06–1.35)	1.2 (1.06–1.35)	8.4	0.26 (−0.15)	1.2 (1.06)
	fall	217	0.69 (0.6–0.78)	0.69 (0.6–0.79)	30.84	−0.54 (−0.99)	0.69 (0.6)
	Product dose omission issue	184	0.76 (0.66–0.88)	0.76 (0.66–0.88)	13.15	−0.39 (−0.87)	0.77 (0.66)
	Product dose omission issue	148	0.77 (0.66–0.91)	0.77 (0.66–0.91)	9.79	−0.37 (−0.91)	0.77 (0.66)
	Inappropriate schedule of product administration	143	1.52 (1.29–1.8)	1.52 (1.29–1.79)	25.07	0.6 (0.06)	1.52 (1.29)
	Overdose	133	0.49 (0.41–0.58)	0.49 (0.41–0.58)	70.71	−1.03 (−1.59)	0.49 (0.41)
	Wrong technique in product usage process	108	0.47 (0.39–0.57)	0.47 (0.39–0.57)	63.87	−1.08 (−1.71)	0.47 (0.39)
Investigations	Weight decreased	755	2.97 (2.77–3.2)	2.91 (2.71–3.13)	950.21	1.54 (1.29)	2.9 (2.7)
	Weight increased	461	2.06 (1.87–2.25)	2.04 (1.86–2.23)	242.91	1.02 (0.71)	2.03 (1.85)
	Blood pressure increased	296	2.01 (1.79–2.25)	2 (1.78–2.24)	146.36	0.99 (0.61)	1.99 (1.78)
	C-reactive protein increased	186	6.88 (5.95–7.96)	6.84 (5.92–7.9)	907.92	2.75 (2.27)	6.75 (5.83)
	Haemoglobin decreased	186	1.96 (1.7–2.27)	1.95 (1.69–2.25)	85.48	0.96 (0.48)	1.95 (1.69)
	Heart rate decreased	150	4.56 (3.88–5.35)	4.53 (3.86–5.32)	405.9	2.17 (1.63)	4.5 (3.83)
	Heart rate increased	150	1.51 (1.29–1.77)	1.51 (1.28–1.77)	25.11	0.59 (0.06)	1.51 (1.28)
	Product residue present	148	26.51 (22.44–31.31)	26.35 (22.33–31.09)	3,378.6	4.64 (4.09)	24.89 (21.07)
	Hepatic enzyme increased	116	1.8 (1.5–2.16)	1.8 (1.5–2.16)	40.12	0.84 (0.24)	1.79 (1.49)
	Drug level decreased	101	12.64 (10.37–15.41)	12.59 (10.34–15.34)	1,036.41	3.62 (2.96)	12.26 (10.06)
Metabolism and nutrition disorders	Decreased appetite	297	1.45 (1.29–1.62)	1.44 (1.29–1.61)	40.05	0.53 (0.14)	1.44 (1.28)
	Dehydration	242	1.99 (1.75–2.26)	1.98 (1.75–2.25)	116.94	0.98 (0.56)	1.98 (1.74)
	Respiratory failure	103	1.39 (1.14–1.68)	1.39 (1.14–1.68)	10.65	0.47 (−0.17)	1.38 (1.14)
Musculoskeletal and connective tissue disorders	Arthralgia	817	2.24 (2.09–2.4)	2.2 (2.06–2.35)	539.26	1.13 (0.9)	2.19 (2.05)
	Pain in extremity	325	1.1 (0.98–1.22)	1.09 (0.98–1.22)	2.63	0.13 (−0.24)	1.1 (0.98)
	Back pain	322	1.44 (1.29–1.6)	1.43 (1.28–1.59)	41.49	0.51 (0.15)	1.43 (1.28)
	Muscle spasms	304	1.63 (1.45–1.82)	1.62 (1.45–1.81)	71.83	0.69 (0.32)	1.62 (1.44)
	Myalgia	295	1.62 (1.44–1.82)	1.61 (1.44–1.8)	67.96	0.69 (0.3)	1.61 (1.43)
	Arthritis	223	3.06 (2.68–3.49)	3.04 (2.66–3.46)	301.56	1.6 (1.16)	3.02 (2.65)
	Chills	204	1.86 (1.62–2.13)	1.85 (1.61–2.12)	79.18	0.89 (0.43)	1.85 (1.61)
	Joint swelling	155	1.47 (1.25–1.72)	1.47 (1.25–1.71)	22.46	0.55 (0.02)	1.46 (1.25)
	Fistula	149	17.03 (14.45–20.07)	16.93 (14.38–19.93)	2,134.31	4.03 (3.48)	16.33 (13.86)
	Musculoskeletal stiffness	119	1.42 (1.19–1.7)	1.42 (1.19–1.7)	14.41	0.51 (−0.09)	1.42 (1.19)
Nervous system disorders	Headache	940	1.44 (1.35–1.54)	1.42 (1.34–1.52)	121.24	0.51 (0.29)	1.42 (1.33)
	Tremor	140	0.76 (0.64–0.9)	0.76 (0.65–0.9)	10.22	−0.39 (−0.94)	0.76 (0.65)
	Somnolence	138	0.62 (0.53–0.74)	0.62 (0.53–0.74)	30.92	−0.68 (−1.23)	0.63 (0.53)
	Vision blurred	120	0.83 (0.7–1)	0.83 (0.7–0.99)	3.92	−0.26 (−0.86)	0.83 (0.7)
	Muscular weakness	102	0.9 (0.74–1.1)	0.9 (0.74–1.1)	0.96	−0.15 (−0.79)	0.9 (0.74)
Pregnancy, puerperium and perinatal conditions	Maternal exposure during pregnancy	193	2.23 (1.94–2.58)	2.22 (1.93–2.56)	128.65	1.15 (0.68)	2.22 (1.92)
	Foetal exposure during pregnancy	188	1.77 (1.54–2.05)	1.77 (1.53–2.04)	62.01	0.82 (0.34)	1.77 (1.53)
	Exposure during pregnancy	150	2.35 (2–2.76)	2.34 (1.99–2.74)	113.17	1.22 (0.69)	2.33 (1.98)
	Exposure during pregnancy	103	1.63 (1.34–1.98)	1.63 (1.34–1.97)	24.17	0.7 (0.06)	1.62 (1.34)
	Premature baby	101	2.43 (2–2.96)	2.42 (2–2.95)	82.81	1.27 (0.63)	2.42 (1.99)
Product issues	Product quality issue	106	0.63 (0.52–0.76)	0.63 (0.52–0.76)	22.68	−0.66 (−1.29)	0.63 (0.52)
	Device malfunction	104	1.63 (1.34–1.98)	1.63 (1.34–1.97)	24.48	0.7 (0.06)	1.62 (1.34)
Psychiatric disorders	Depression	315	1.16 (1.03–1.29)	1.16 (1.03–1.29)	6.46	0.21 (−0.16)	1.15 (1.03)
	Anxiety	283	0.82 (0.73–0.92)	0.82 (0.73–0.92)	11.1	−0.29 (−0.68)	0.82 (0.73)
	Insomnia	228	0.77 (0.68–0.88)	0.78 (0.68–0.88)	14.65	−0.36 (−0.8)	0.78 (0.68)
	Stress	168	2.41 (2.07–2.81)	2.4 (2.07–2.8)	136.07	1.26 (0.76)	2.4 (2.06)
	Confusional state	121	0.7 (0.58–0.83)	0.7 (0.58–0.83)	15.75	−0.52 (−1.11)	0.7 (0.58)
Renal and urinary disorders	Urinary tract infection	210	1.41 (1.23–1.62)	1.41 (1.23–1.61)	24.66	0.49 (0.04)	1.41 (1.23)
	Renal failure	163	0.98 (0.84–1.14)	0.98 (0.84–1.14)	0.07	−0.04 (−0.55)	0.98 (0.84)
	Acute kidney injury	142	0.93 (0.79–1.1)	0.93 (0.79–1.1)	0.64	−0.1 (−0.65)	0.93 (0.79)
	Tubulointerstitial nephritis	131	6.14 (5.16–7.29)	6.11 (5.14–7.25)	547.18	2.59 (2.02)	6.04 (5.08)
	Nephrolithiasis	128	3.06 (2.57–3.64)	3.05 (2.56–3.63)	173.18	1.6 (1.02)	3.03 (2.55)
	Chronic kidney disease	127	1.02 (0.86–1.22)	1.02 (0.86–1.21)	0.04	0.03 (−0.55)	1.02 (0.86)
	Renal impairment	101	1.16 (0.95–1.41)	1.16 (0.95–1.4)	1.97	0.21 (−0.44)	1.16 (0.95)
Respiratory, thoracic and mediastinal disorders	Dyspnoea	584	1.04 (0.96–1.13)	1.04 (0.96–1.13)	0.88	0.06 (−0.22)	1.04 (0.96)
	Pneumonia	411	1.43 (1.3–1.58)	1.43 (1.3–1.57)	52.51	0.51 (0.18)	1.43 (1.29)
	Cough	396	1.59 (1.44–1.76)	1.58 (1.44–1.75)	85.1	0.66 (0.33)	1.58 (1.43)
	Nasopharyngitis	284	1.77 (1.57–1.99)	1.76 (1.57–1.98)	92.72	0.81 (0.42)	1.76 (1.56)
	COVID-19	257	2.14 (1.89–2.42)	2.13 (1.88–2.4)	152.23	1.08 (0.67)	2.12 (1.87)
	Sinusitis	198	2.21 (1.92–2.55)	2.2 (1.92–2.53)	128.81	1.14 (0.67)	2.2 (1.91)
	Asthma	150	1.52 (1.29–1.78)	1.51 (1.29–1.78)	25.76	0.6 (0.06)	1.51 (1.29)
	Rhinorrhoea	128	2.18 (1.83–2.6)	2.18 (1.83–2.59)	80.12	1.12 (0.54)	2.17 (1.82)
	Pleural effusion	119	2.03 (1.69–2.43)	2.02 (1.69–2.42)	60.44	1.01 (0.42)	2.02 (1.69)
	Nasal congestion	114	2.2 (1.83–2.65)	2.2 (1.83–2.64)	73.06	1.13 (0.52)	2.19 (1.82)
	Bronchitis	103	1.53 (1.26–1.86)	1.53 (1.26–1.85)	18.27	0.61 (−0.03)	1.53 (1.26)
Skin and subcutaneous tissue disorders	Rash	526	1.25 (1.15–1.37)	1.25 (1.15–1.36)	26.18	0.32 (0.03)	1.25 (1.14)
	Pruritus	329	1.02 (0.92–1.14)	1.02 (0.92–1.14)	0.11	0.03 (−0.34)	1.02 (0.91)
	Alopecia	317	1.52 (1.36–1.7)	1.52 (1.36–1.69)	55.74	0.6 (0.23)	1.52 (1.36)
	Paraesthesia	220	1.35 (1.18–1.54)	1.35 (1.18–1.54)	19.42	0.43 (−0.01)	1.35 (1.18)
	Hypoaesthesia	219	1.42 (1.24–1.62)	1.41 (1.24–1.61)	26.19	0.5 (0.05)	1.41 (1.24)
	Erythema	211	1.39 (1.22–1.6)	1.39 (1.21–1.59)	22.82	0.47 (0.02)	1.39 (1.21)
	Injection site erythema	196	1.41 (1.23–1.62)	1.41 (1.22–1.62)	22.75	0.49 (0.02)	1.41 (1.22)
	Night sweats	156	5.44 (4.64–6.38)	5.41 (4.62–6.33)	550.5	2.42 (1.9)	5.36 (4.57)
	Hyperhidrosis	129	0.95 (0.8–1.13)	0.95 (0.8–1.13)	0.3	−0.07 (−0.65)	0.95 (0.8)
	Lupus-like syndrome	113	14.11 (11.69–17.02)	14.04 (11.65–16.93)	1,313.7	3.77 (3.15)	13.63 (11.3)
Surgical and medical procedures	Hospitalisation	132	0.79 (0.66–0.94)	0.79 (0.67–0.94)	7.29	−0.34 (−0.91)	0.79 (0.67)
Vascular disorders	Haemorrhage	290	2.45 (2.18–2.76)	2.44 (2.17–2.73)	243.99	1.28 (0.89)	2.43 (2.16)
	Hypertension	284	1.37 (1.22–1.54)	1.36 (1.21–1.53)	27.06	0.44 (0.05)	1.36 (1.21)
	Migraine	200	2.15 (1.87–2.47)	2.14 (1.86–2.46)	119.93	1.09 (0.63)	2.13 (1.85)
	Hypotension	193	0.98 (0.85–1.12)	0.98 (0.85–1.12)	0.1	−0.04 (−0.51)	0.98 (0.85)
	Injection site haemorrhage	160	1.88 (1.61–2.2)	1.87 (1.6–2.19)	64.18	0.9 (0.38)	1.87 (1.6)
	Pulmonary embolism	130	1.14 (0.96–1.35)	1.14 (0.96–1.35)	2	0.18 (−0.39)	1.14 (0.96)
	Blood pressure fluctuation	129	7.52 (6.31–8.95)	7.48 (6.29–8.9)	706.31	2.88 (2.3)	7.37 (6.19)
	Contusion	116	1.26 (1.05–1.52)	1.26 (1.05–1.51)	6.08	0.34 (−0.27)	1.26 (1.05)
	Injection site haematoma	108	4.15 (3.43–5.02)	4.14 (3.42–5)	251.56	2.04 (1.41)	4.11 (3.4)

**Abbreviations**: ROR, reporting odds ratio; CI, confidence interval; PRR, proportional reporting ratio; χ2, chi-squared; IC, information component; EBGM, empirical Bayesian geometric mean.

Signal strengths and reports of mesalazine at the SOC level (PT > 100) were described in Table 4. Statistically, we found that mesalazine -induced AEs occurrence targeted 27 SOCs. The significant SOCs were “Gastrointestinal disorders (SOC: 10017947)”, “blood and lymphatic system disorders (SOC: 10005329)”, “Infections and infestations (SOC: 10021881)” and “Pregnancy, puerperium and perinatal conditions (SOC: 10036585)”.

**TABLE 4:** Signal Strength of Reports of mesalazine at the Preferred Terms Level in FAERS Database.

System organ class (SOC)	Cases reporting SOC	ROR (95% two-sided CI)	PRR (95% two-sided CI)	χ^2^	IC (IC025)	EBGM (EBGM05)
Blood and lymphatic system disorders	2,157	2.03 (1.94–2.11)	2.02 (1.94–2.11)	1,113.96	1.01 (0.87)	2.02 (1.94)
Cardiac disorders	3,400	1.14 (1.1–1.18)	1.14 (1.1–1.18)	58.25	0.19 (0.07)	1.14 (1.1)
Congenital, familial and genetic disorders	182	1.99 (1.72–2.31)	1.99 (1.72–2.31)	89.7	0.99 (0.51)	1.99 (1.72)
Ear and labyrinth disorders	267	1.24 (1.1–1.4)	1.24 (1.1–1.4)	12.13	0.31 (−0.09)	1.24 (1.1)
Endocrine disorders	383	0.76 (0.69–0.84)	0.76 (0.69–0.84)	29.5	−0.4 (−0.73)	0.76 (0.69)
Eye disorders	999	1.27 (1.19–1.35)	1.27 (1.19–1.35)	57.49	0.34 (0.14)	1.27 (1.19)
Gastrointestinal disorders	17,643	3.28 (3.23–3.33)	3.28 (3.23–3.32)	27,695.53	1.7 (1.65)	3.26 (3.21)
General disorders and administration site conditions	15,215	1.39 (1.36–1.41)	1.38 (1.36–1.41)	1,619.74	0.47 (0.41)	1.38 (1.36)
Hepatobiliary disorders	1,109	1.8 (1.7–1.91)	1.8 (1.7–1.91)	392.59	0.85 (0.65)	1.8 (1.69)
Immune system disorders	2,555	1.58 (1.52–1.64)	1.58 (1.52–1.64)	543.52	0.66 (0.53)	1.58 (1.52)
Infections and infestations	3,308	2.63 (2.54–2.72)	2.63 (2.54–2.72)	3,323.34	1.39 (1.27)	2.62 (2.53)
Injury, poisoning and procedural complications	7,161	1.28 (1.25–1.31)	1.28 (1.25–1.31)	444.71	0.36 (0.28)	1.28 (1.25)
Investigations	6,634	1.92 (1.87–1.96)	1.91 (1.87–1.96)	2,886.29	0.93 (0.85)	1.91 (1.87)
Metabolism and nutrition disorders	1,671	1.44 (1.38–1.52)	1.44 (1.38–1.52)	227.77	0.53 (0.37)	1.44 (1.38)
Musculoskeletal and connective tissue disorders	4,458	1.51 (1.47–1.56)	1.51 (1.47–1.56)	774.74	0.6 (0.5)	1.51 (1.47)
Neoplasms benign, malignant and unspecified (incl cysts and polyps)	697	0.73 (0.68–0.78)	0.73 (0.68–0.78)	70.62	−0.46 (−0.71)	0.73 (0.68)
Nervous system disorders	3,537	0.82 (0.79–0.84)	0.82 (0.79–0.84)	146.38	−0.29 (−0.4)	0.82 (0.79)
Pregnancy, puerperium and perinatal conditions	1,456	2.21 (2.1–2.33)	2.21 (2.1–2.33)	958.67	1.14 (0.97)	2.2 (2.09)
Psychiatric disorders	2,699	0.65 (0.63–0.68)	0.65 (0.63–0.68)	505.55	−0.62 (−0.75)	0.65 (0.63)
Renal and urinary disorders	2028	1.32 (1.26–1.37)	1.31 (1.26–1.37)	152.56	0.39 (0.25)	1.31 (1.26)
Reproductive system and breast disorders	676	0.89 (0.82–0.96)	0.89 (0.82–0.96)	9.58	−0.17 (−0.42)	0.89 (0.82)
Respiratory, thoracic and mediastinal disorders	5,003	1.63 (1.58–1.67)	1.63 (1.58–1.67)	1,207.49	0.7 (0.61)	1.63 (1.58)
Skin and subcutaneous tissue disorders	4,218	1.43 (1.39–1.47)	1.43 (1.38–1.47)	538.05	0.51 (0.41)	1.43 (1.38)
Social circumstances	420	1.51 (1.37–1.66)	1.51 (1.37–1.66)	72.73	0.6 (0.27)	1.51 (1.37)
Surgical and medical procedures	939	1.36 (1.28–1.45)	1.36 (1.28–1.45)	90.62	0.45 (0.23)	1.36 (1.28)
Vascular disorders	2,914	1.43 (1.38–1.48)	1.43 (1.38–1.48)	371.21	0.51 (0.39)	1.43 (1.37)
Product issues	772	0.86 (0.8–0.92)	0.86 (0.8–0.92)	18.19	−0.22 (−0.46)	0.86 (0.8)

**Abbreviations**: SOC, system organ class; ROR, reporting odds ratio; CI, confidence interval; PRR, proportional reporting ratio; χ2, chi-squared; IC, information component; EBGM, empirical Bayesian geometric mean. The PTs, in neoplasms benign, malignant and unspecified (incl cysts and polyps) are not included because the majority of indications are cancers.

### 3.3 Time to onset of mesalazine-associated adverse events

**FIGURE 2:**
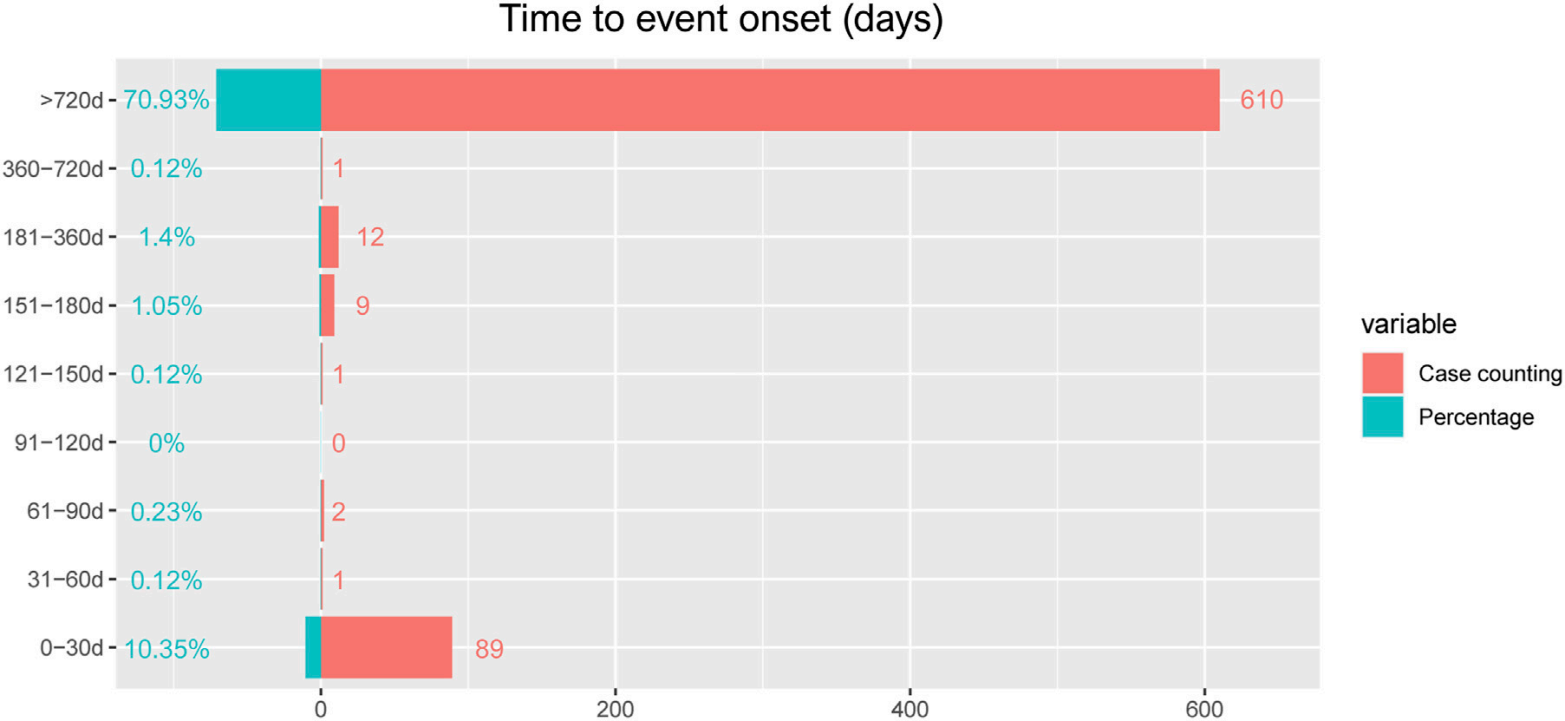
Time to event onset times.

The onset times of mesalazine-associated AEs were extracted from the database. Excluding inaccurate, missing or unknown onset time reports, a total of 725 mesalazine -associated AEs reported onset time and the median onset time was 1,127 days (interquartile range [IQR] 1,127–1,164 days). As Figure 2 illustrated, results indicated that most of the AE cases occurred 2 years later (n = 610, 70.93%) and within the first 1 month (n = 89, 10.35%) after mesalazine initiation.

## 4 Conclusion

In conclusion, the present study using pharmacovigilance analysis of FAERS database scientifically and systematically quantified the potential risks, time to AE onsets and the safety signal spectrum with mesalazine treatment. Unexpected and new significant AEs as Dizziness, Drug ineffective, Drug hypersensitivity, Infection, Off label use, Weight decreased, Decreased appetite, Arthralgia, Headache, Dyspnoea, Rash AEs are frequent AEs for which patients should be monitored. Our study could provide valuable evidence for further studies and clinical practice of mesalazine.

## 5 Discussion

To the best of our knowledge, this is the first most comprehensive and systematic pharmacovigilance study on mesalazine-associated AEs by post-marketing based on the FAERS database. We presented a more accurate and detailed description and characterization of mesalazine-associated AEs to date. However, because results of the low response rate in active ulcerative colitis and in Crohn’s disease treatment, some AEs such as gastrointestinal disorders and infections and infestations might be considered as the signs of ineffectiveness rather than real adverse events. In the cases with mesalazine-associated AEs, the proportion of female is 50.34% slightly higher than that of males (39.32%). However, based on the unknown cases (10.34%), we cannot compare credible gender ratios in these cases. Almost half of the reports (49.94%) were submitted by health professionals, which might be considered a more reliable source of reporting. Reports of AEs since 2018 have continued to increase due to the widespread use and increased awareness of healthcare professionals, strongly underling the need for constant epidemiological surveillance (Singh et al., 2015). In our study, mesalazine demonstrated a slightly higher AEs proportion in patients aged in elderly individuals (aged >68 years) and patients aged 18–28 years, although the difference among all different ages is so small. Besides, a large number of cases with unknown ages also results in the difficulty in the discovery of the ages of patients which is most easily to occur mesalazine-associated AEs.

According to the disproportionality analysis, the most common and significant SOCs such as “Gastrointestinal disorders”, “blood and lymphatic system disorders”, “Infections and infestations”, and “Pregnancy, puerperium and perinatal conditions” were consistent with the safety data in the label and clinical trials. Among the SOC of Gastrointestinal disorders, AEs with the highest number of reports were colitis ulcerative, diarrhoea, crohn’s disease, abdominal pain, nausea. Blood and lymphatic system disorders includes anaemia, pancytopenia, thrombocytopenia, leukopenia.

Notably, long-term use of mesalazine is associated with a risk of skin AEs, and the most common is rash. However, early study provided experimental evidence that oral mesalazine treatment is effective to prevent cutaneous fibrosis development in genetically predisposed mice. Because lossing of PLK2 function induces spontaneous fibrotic remodeling of the skin due to aberrant myofibroblast activation and collagen accumulation (Newe et al., 2021; Xie et al., 2022).

The mesalazine are generally well tolerated; headache, nausea, diarrhea and abdominal pain are the most common, still very rare side effects (Zhang et al., 2019). Nephrotoxicity is extremely rare in patients on 5-ASA medications, with a mean incidence of 0.3% per person-year (Bonovas et al., 2019; Castro Tejera et al., 2022). In most cases, renal failure is caused by an acute or chronic interstitial nephritis, which is idiosyncratic and unrelated to 5-ASA formulation and dose (Gisbert et al., 2007; Yang et al., 2014). Despite its rarity, nephrotoxicity has to be taken into account in all patients treated with mesalazine, and scheduled controls of renal function are suggested in IBD treatment guidelines (Nardelli et al., 2017; Nguyen et al., 2018).

Results of this study indicated that the median onset time was 1,127 days, and most of the cases occurred 2 years later (n = 610, 70.93%) and within the first 1 month (n = 89, 10.35%) after mesalazine initiation. This may be due to the existence of a latency period of the drug, or the patient’s body resistance, slow metabolism, and prolonged duration required for cumulative drug concentration build-up, the body is unable to fully absorb the drug, so most people’s adverse drug reaction appears later. Therefore, a longer follow-up period is needed to observe the ADRs of mesalazine in future clinical studies.

Despite the advantages of real-world large-sample research and the data mining techniques in this study, there are still some limitations that warrant discussion (Nguyen et al., 2012; Moja et al., 2015). First, FAERS is a spontaneous reporting system with incomplete and incorrect information collected from different countries and professionals, thus the quality might be variable, which may lead to bias in the analysis. Second, multiple unmeasured confounders such as potential drug-drug interactions, comorbidities and drug combinations, which might affect AEs, were not included in the data analysis. Third, despite having access to thousands of case reports, the safety reports do not provide detailed information of patients exposed to the drug without AEs. Moreover, since mesalazine is marketed in some country as a generic drug and this might modify the rate of absorption and thus the safety profile of the drug. Therefore, the true incidence of AEs cannot be determined from FAERS data. Fourth, it was unable to infer an exact causal relationship. The disproportionality analysis neither quantified risk nor existed causality, but only provided an estimation of the signal strength, which was only statistically significant. Prospective clinical studies are still needed to confirm the causal relationship between them. Despite these limitations, our results would provide a valuable reference for healthcare professionals to closely follow-up patients and monitor the associated adverse reactions of mesalazine (Law et al., 2020; Shu et al., 2022a; Shu et al., 2022b).

## Data Availability

The original contributions presented in the study are included in the article/Supplementary Material, further inquiries can be directed to the corresponding authors.
